# Treatment with *Rhodiola crenulata* root extract ameliorates insulin resistance in fructose-fed rats by modulating sarcolemmal and intracellular fatty acid translocase/CD36 redistribution in skeletal muscle

**DOI:** 10.1186/s12906-016-1176-z

**Published:** 2016-07-12

**Authors:** Ting Chen, Ling Yao, Dazhi Ke, Weiguo Cao, Guowei Zuo, Liang Zhou, Jian Jiang, Johji Yamahara, Yuhao Li, Jianwei Wang

**Affiliations:** Faculty of Basic Medical Sciences, Chongqing Medical University, Chongqing, 400016 China; The Laboratory of Traditional Chinese Medicine, Chongqing Medical University, Chongqing, 400016 China; The Second Affiliated Hospital, Chongqing Medical University, Chongqing, 400010 China; College of Laboratory Medicine, Chongqing Medical University, Chongqing, 400016 China; Endocrinology and Metabolism Group, Sydney Institute of Health Sciences/Sydney Institute of Traditional Chinese Medicine, Sydney, NSW 2000 Australia; Pharmafood Institute, Kyoto, 602-8136 Japan

**Keywords:** Fatty acid translocase/CD36, Insulin resistance, *Rhodiola*, Sarcolemma

## Abstract

**Background:**

*Rhodiola* species have been used for asthenia, depression, fatigue, poor work performance and cardiovascular diseases, all of which may be associated with insulin resistance. To disclose the underlying mechanisms of action, the effect of *Rhodiola crenulata* root (RCR) on insulin resistance was investigated.

**Methods:**

Male Sprague-Dawley rats were treated with liquid fructose in their drinking water over 18 weeks. The extract of RCR was co-administered (once daily by oral gavage) during the last 5 weeks. The indexes of lipid and glucose homeostasis were determined enzymatically and/or by ELISA. Gene expression was analyzed by Real-time PCR, Western blot and/or confocal immunofluorescence.

**Results:**

RCR extract (50 mg/kg) suppressed fructose-induced hyperinsulinemia and the increases in the homeostasis model assessment of insulin resistance index and the adipose tissue insulin resistance index in rats. Additionally, this treatment had a trend to restore the ratios of glucose to insulin and non-esterified fatty acids (NEFA) to insulin. Mechanistically, RCR suppressed fructose-induced acceleration of the clearance of plasma NEFA during oral glucose tolerance test (OGTT), and decreased triglyceride content and Oil Red O staining area in the gastrocnemius. Furthermore, RCR restored fructose-induced sarcolemmal overexpression and intracellular less distribution of fatty acid translocase/CD36 that contributes to etiology of insulin resistance by facilitating fatty acid uptake.

**Conclusion:**

These results suggest that RCR ameliorates insulin resistance in fructose-fed rats by modulating sarcolemmal and intracellular CD36 redistribution in the skeletal muscle. Our findings may provide a better understanding of the traditional use of *Rhodila* species.

## Background

Insulin resistance is the thread that runs through many chronic afflictions of modern times**–**obesity, cardiovascular disease, and most conspicuously, type 2 diabetes [1]. Recently, a large body of evidence has suggested that depression and fatigue are linked to some metabolic disorders, such as obesity, insulin resistance, diabetes and liver disease [2–9]. Depression may occur as a consequence of having diabetes. It has been suggested that insulin resistance is a part of the pathophysiology of affective disorders, and that improvement of insulin resistance may reduce the severity of depressive symptoms [10, 11]. Treatments of type 2 diabetic patients with insulin sensitizers rosiglitazone and pioglitazone improve not only metabolic derangements, but also affective disorders [10, 12–14]. On the other hand, depression may also be a risk factor for the onset of type 2 diabetes; depressed adults have a 37 % increased risk of developing type 2 diabetes mellitus [15]. Similarly, endocrine dysfunction is a common etiology of fatigue. Chronic fatigue has been associated with obesity and its metabolic complications [16]. Therefore, these research findings suggest that amelioration of insulin resistance may result in improvement of depression and fatigue, and conversely, effective prevention or treatment of depression may reduce insulin resistance-associated health consequences.

The roots of the alpine plant genus *Rhodiola* (Crassulaceae) have been traditional medicines in Eastern Europe and Asia. *Rhodila* species are used as tonics and stimulants to increase physical endurance, work productivity and longevity, and to enhance energy levels [17, 18]. Thus, *Rhodila* species are used to treat patients with asthenia [17, 18]. It has been demonstrated that treatment of type 2 diabetic patients with *Rhodiola crenulata* tea for 12–24 months significantly lowered blood glucose concentration, accompanied by improvement of dysfunctions of liver and kidneys [19]. *Rhodioa rosea*, another species of *Rhodiola*, has been reported to show synergistic effects with losartan, an angiotensin II type 1 receptor blocker, on hyperglycemia and hyperlipidemia in patients with early diabetic nephropathy [20]. We have also demonstrated that treatment with RCR ameliorates derangements of glucose and lipid metabolism in Zucker diabetic fatty rats [21]. Salidroside, one of the prominent active components contained in *Rhodila* species, ameliorates insulin resistance in db/db mice [22]. In addition, *Rhodila* species have also been used to treat fatigue and depression [17, 18]. Based on the research findings, we hypothesized that the underlying mechanisms of action of *Rhodila* species for their traditional use were associated with improvement of insulin resistance.

Strong evidence suggests that chronically high consumption of fructose in rodents leads to dyslipidemia, fatty liver, insulin resistance, obesity, and type 2 diabetes mellitus [23]. During obesity an increase in the circulating fatty acids released from the abdominal fat depots leads to increased uptake by non-adipose tissues, such as skeletal muscle, thereby inhibiting glucose oxidation and reducing insulin sensitivity [24]. Thus, abnormal fatty acid metabolism, especially in skeletal muscle, is linked to insulin resistance [25]. Membrane uptake of long-chain fatty acids is the first step in cellular fatty acid utilization and a point of metabolic regulation. Fatty acid translocase/CD36, a multi-functional glycoprotein, facilitates a major fraction of fatty acid uptake by some key tissues, such as skeletal muscle, and plays an important role in membrane transport and utilization of long-chain fatty acids [26]. CD36 is involved in a number of metabolic pathways, and contributes to development of insulin resistance and the metabolic syndrome [25, 26]. Research has shown that sugar-sweetened nonalcoholic beverages, such as soft drinks, appear as the major source of fructose for all classes of age considered, except for children younger than 6 years and adults older than 50 years [23]. To better understand the traditional application of *Rhodila* species, the present study investigated the effect of an extract of RCR on insulin resistance and the possible involvement of CD36 in the underlying mechanisms of action in liquid fructose-fed rats.

## Methods

### Preparation and identification of an aqueous-ethanolic extract of RCR

**Fig. 1:**
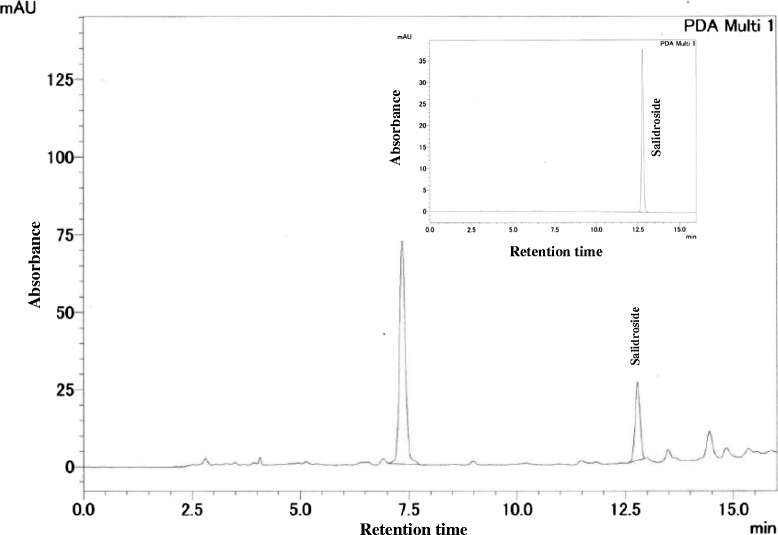
HPLC fingerprints of the aqueous-ethanolic extract of *Rhodiola crenulata* root (RCR) and salidroside standard (inset) at 275 nm

RCR was collected in Tibet, China. A voucher specimen (No: PS0183) was deposited in Pharmafood Institute, Kyoto, Japan. Dried RCR was identified botanically by Professor Johji Yamahara, who is an expert in taxonomy. The aqueous-ethanolic extract used in the present study was prepared. Briefly, dried RCR materials were ground into crude powder, immersed in 7 volumes of 50 % ethanol (50 °C) with intermittent shaking for 5 h, and filtrated. The residue was extracted for additional 2 times using the same method. The combined filtrate was evaporated under reduced pressure below 45 °C. The yield of the extract was 15 %. The extract was quantified by the HPLC method previously described [21] to contain 2.5 % salidroside, one of prominent active components contained in *Rhodila* species. HPLC fingerprints of RCR extract and salidroside standard (inset) at 275 nm are shown in Fig. 1.

### Animals, diet and experimental protocol

All experimental procedures were carried out in accordance with the internationally accepted principles for laboratory animal use and care, and approved by the Animal Ethics Committee, Chongqing Medical University, China.

Male Sprague–Dawley rats weighing 210–230 g and the standard chow were supplied by the laboratory animal center, Chongqing Medical University, China. Rats were housed in a temperature controlled facility (21 ± 1 °C, 55 ± 5 % relative humidity) with a 12-h light/dark cycle. Animals were allowed free access to water and the standard chow for at least 1 week prior to starting the experiments.

Fructose in drinking water used for the present study, has been described previously [27–31]. Thirty-three rats were divided initially into 2 groups: water control free access to water (*n* = 6), and fructose group free access to 10 % fructose solution (w/v, preparation every day) (*n* = 27). This fructose group was further divided into the following 3 groups (*n* = 9) 13 weeks after study commencement and had continued free access to 10 % fructose solution until the end of week 18: fructose control, fructose RCR 10 mg/kg and fructose RCR 50 mg/kg. Animals in RCR-treated groups were administered RCR extract at the dosages of 10 and 50 mg/kg (suspended in 5 % Gum Arabic solution, gavage once daily), respectively. The rats in the corresponding water- and fructose-control groups received vehicle (5 % Gum Arabic) alone. All rats had free access to the standard chow. To avoid stress and increase monitoring accuracy of fructose and chow intakes, only 2–3 rats were housed in a cage at any given time. The consumed chow and fructose solution were measured daily and the intake of fructose was calculated. At the end of week 17, blood samples were collected by retroorbital venous puncture under ether anesthesia under overnight-fasted condition. Here, the plasma concentrations of glucose (kit from Kexin Institute of Biotechnology, Shanghai, China), insulin (kit from Morinaga Biochemical Industries, Tokyo, Japan), triglyceride (Triglyceride-E kit, Wako, Osaka, Japan), non-esterified fatty acids (NEFA) (NEFA-C kit, Wako, Osaka, Japan) and total cholesterol (kit from Kexin Institute of Biotechnology, Shanghai, China) were determined using enzymatic methods or by ELISA. Immediately followed, OGTT was performed. Animals were weighed and euthanized after being fasted overnight at the end of week 18. Epididymal fat and gastrocnemius (right) were collected. Segments of gastrocnemius were individually snap frozen in liquid nitrogen and stored at -80 °C for subsequent determination of the content of triglyceride and/or gene expression.

### OGTT

After being fasted overnight, all rats received a glucose solution (2 g/kg in 5 ml) by the oral gavage. Blood samples were collected prior to and 20, 60 and 120 min after administration of glucose solution. Plasma concentrations of glucose and NEFA were determined. The homeostasis model assessment of insulin resistance (HOMA-IR) index {[fasted insulin (μIU/mL) × fasted glucose (mM)]/22.5} and the Adipo-IR index [Adipo-IR index = fasted insulin (mmol/L) × fasted NEFA (pmol/L)] were calculated [32, 33]. The clearance of plasma NEFA was calculated as the following formula: the concentration under fasted condition (0 min) – the concentration at the time-point (20, 60 or 120 min) after glucose administration. The area under the curve (AUC) of plasma concentrations and/or clearances of glucose and NEFA was calculated, respectively.

### Determination of triglyceride content in skeletal muscle

Triglyceride content in the gastrocnemius was determined as described previously [28, 31]. Briefly, 100 mg of tissue was homogenized and extracted with 2 mL of isopropanol. After centrifugation (625 × g), the triglyceride content in supernatant was determined enzymatically (Wako, Osaka, Japan).

### Measurement of fatty droplet accumulation in skeletal muscle

A portion of gastrocnemius was frozen, and six-micron sections were cut and stained with Oil Red O for examination of fatty droplet accumulation (BX-51, Olympus Corporation, Tokyo, Japan). Forty fields in individual section were randomly selected, and the Oil Red O-stained and total fiber areas were measured using an ImageJ 1.43 analyzing system. The ratio of the Oil Red O-stained area to the total tissue area was calculated (%).

### Real time PCR

Real time PCR was performed as described previously [31]. Total RNA was isolated from gastrocnemius of individual rats using TRIzol (Takara, Dalian, China). cDNA was synthesized using M-MLV RTase cDNA Synthesis Kit (Takara, Dalian, China) according to the manufacturer’s instructions. Real time PCR was performed with the CFX 96 Real Time PCR Detection System (Biorad Laboratories Inc, Hercules, CA, USA) using the SYBR® Premix Ex Taq™ II (Takara, Dalian, China). The sequences of primers are shown in Table 1. The gene expression from each sample was analysed in duplicates and normalized against the internal control gene β-actin. Levels in control rats were arbitrarily assigned a value of 1.

**Table 1:** Primer sequences for real time PCR assays

Gene	Accession number	Primer Sequences
β-actin	NM_031144.2	Forward: ACGGTCAGGTCATCACTATCG
		Reverse: GGCATAGAGGTCTTTACGGATG
Adiponectin	NM_144744.3	Forward: CGTTCTCTTCACCTACGACCAGT
		Reverse: ATTGTTGTCCCCTTCCCCATAC
CD36	NM_001109218	Forward: AACCCAGAGGAAGTGGCAAAG
		Reverse: GACAGTGAAGGCTCAAAGATGG
DGAT-1	NM_053437.1	Forward: GGACAAAGACCGGCAGACCA
		Reverse: CAGCATCACCACGCACCAAT
DGAT-2	NM_001012345.1	Forward: CCTGGCAAGAACGCAGTCAC
		Reverse: GAGCCCTCCTCAAAGATCACC
PPAR-γ	AB_011365.1	Forward: GCCCTTTGGTGACTTTATGGAG
		Reverse: GCAGCAGGTTGTCTTGGATGT

Sequences: 5’ to 3’

### Western blot

Western blot was performed basically as described previously [28, 31]. Total protein was prepared from gastrocnemius using the T-PER tissue protein extraction reagent kits (Pierce Biotechnology, Rockford, IL, USA), according to the manufacturer’s instructions. Protein concentration was determined using the Bradford method (Bio Rad Laboratories, Hercules, CA, USA) using bovine serum albumin as a standard. Protein (30 μg) was subjected to SDS-PAGE analysis on a 10 % gel, then electrotransferred onto Polyvinylidene Fluoride Membrane (Amersham, Buckinghamshire, UK). CD36 (dilution 1:1000, Abcam, Cambridge, Massachusetts, USA) was detected with a rabbit polyclonal antibody. Detection of signal was performed using the ECL Western blot detection kit (Pierce Biotechnology, Rockford, IL, USA) with anti-rabbit horseradish peroxidase-conjugated IgG (dilution 1:5,000, Santa Cruz Biotechnology, Santa Cruz, CA, USA) as second antibody, respectively. Polyclonal rabbit β-actin antibody (Cell Signaling Technologies, Beverly, MA, USA) was used as loading control to normalize the signal obtained for CD36 protein. The immunoreactive bands were visualized by autoradiography and the density was evaluated using ImageJ 1.43. Levels in control rats were arbitrarily assigned a value of 1.

### Immunofluorescence staining

To examine CD36 distribution in rat skeletal muscle fibers, cryosections were immunofluorescently labeled and analyzed by confocal microscopy. Transverse cryosections from gastrocnemius were transferred to glass slides, and allowed to dry at room temperature. The sections were blocked with normal goat serum for 30 min and incubated with rabbit polyclonal anti-CD36 antibody (dilution 1:200, Abcam, Cambridge, Massachusetts, USA) in blocking buffer at 4 °C overnight. Sections were rinsed with PBS three times and incubated with CY3-labeled goat anti-rabbit IgG as secondary antibody in blocking buffer for 30 min. Sections were rinsed with PBS three times again and nuclei were counterstained with DAPI (Molecular Probes/Invitrogen Life Technologies, Carlsbad, CA, USA). Finally, sections were mounted and analyzed as described previously [34]. Images were collected with confocal microscope (A1 + R confocal microscope, Nikon Corporation, Tokyo, Japan). Imaging settings were set so that no signal was detected in the respective negative controls and a low fraction of pixels showed saturation intensity values when imaging the stained samples.

### Semi-quantification of CD36 expression [34]

On transverse cryosections from gastrocnemius stained with anti-CD36, skeletal muscle fiber was rated 1, 2 or 3 based on the intensity of the sarcolemmal CD36 fluorescent signal by a person who was blinded. To check interobserver variability, another blinded person also rated the intensity of the fluorescence signal with similar results. In each sample, ~100 fibers were rated. The percentage of fibers rated 1, 2, and 3 each sample was calculated respectively.

### Data analysis

All results are expressed as mean ± SEM. Data were analyzed by ANOVA using StatView, and followed by Student-Newman-Keuls testing to locate the differences between groups. *P* < 0.05 was considered to be statistically significant.

## Results

### Effects on intakes of fructose and chow, body weight increase and epididymal fat weight in rats

**Fig. 2:**
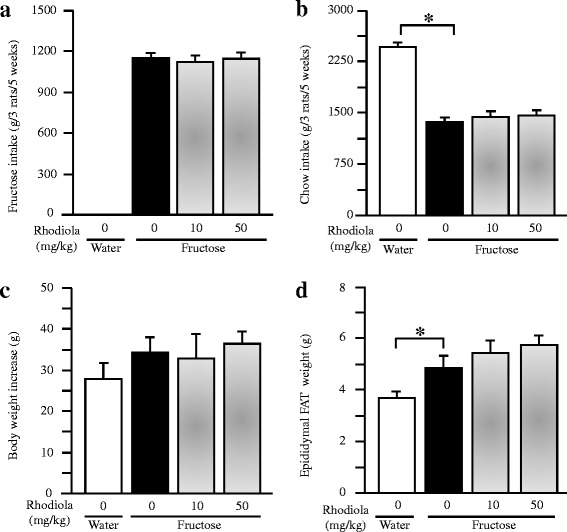
Intakes of fructose (**a**) and laboratory chow (**b**), body weight increase (**c**), and epididymal fat weight (**d**) in rats. The fructose controls (*Rhodiola* 0 mg/kg) and fructose *Rhodiola* (50 mg/kg) group had free access to 10 % fructose in their drinking water over 18 weeks. The water controls (*Rhodiola* 0 mg/kg) had free access to a tap water. *Rhodiola* (50 mg/kg) was administered by gavage daily during the last 5 weeks. The water and fructose controls received vehicle (5 % Gum Arabic) alone. Data are means ± SEM (*n* = 6-9 each group). * *P* < 0.05

Fructose control and fructose RCR groups consumed similar amount of fructose during the last five weeks (Fig. 2a). Intake of fructose decreased chow intake in the same manner in both fructose control and fructose RCR groups (Fig. 2b).

There was no significant difference in body weight increase between water control and fructose control or fructose RCR groups (Fig. 2c). However, long term fructose consumption increased epididymal fat weight, whereas treatment with RCR did not affect epididymal fat weight in fructose-fed rats (Fig. 2d).

### Effects on glucose metabolism in rats

**Fig. 3:**
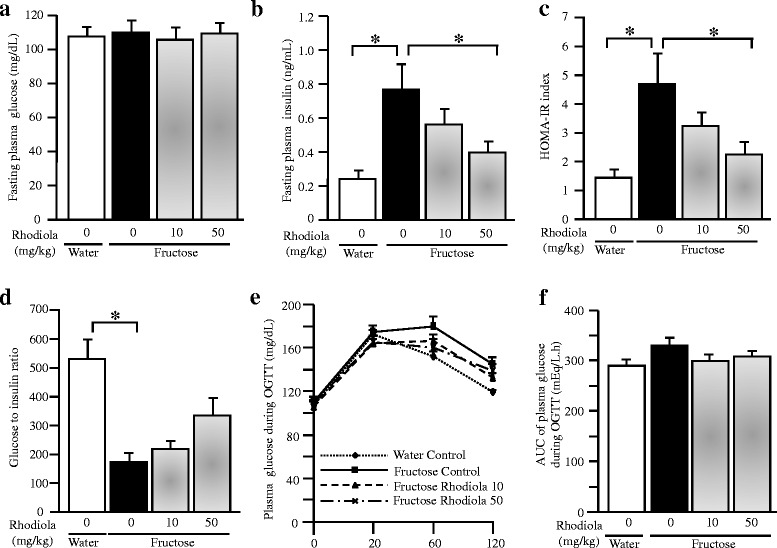
Plasma glucose (**a**) and insulin (**b**) concentrations under fasting condition, HOMA-IR index (**c**), the ratio of glucose to insulin (**d**), plasma glucose concentrations and the AUC of plasma glucose concentrations (**e** and **f**) during OGTT in rats. The fructose controls (*Rhodiola* 0 mg/kg) and fructose *Rhodiola* (50 mg/kg) group had free access to 10 % fructose in their drinking water over 18 weeks. The water controls (*Rhodiola* 0 mg/kg) had free access to a tap water. *Rhodiola* (50 mg/kg) was administered by gavage daily during the last 5 weeks. The water and fructose controls received vehicle (5 % Gum Arabic) alone. Data are means ± SEM (*n* = 6-9 each group). * *P* < 0.05

Although fructose feeding did not alter plasma glucose concentration at the baseline (under fasted condition) (Fig. 3a), it had a trend to increase glucose concentrations during OGTT (Fig. 3e, f). Strikingly, fructose consumption increased basal plasma insulin concentration and the HOMA-IR index by three folds (Fig. 3b, c). Furthermore, fructose feeding decreased the ratio of glucose to insulin (Fig. 3d). RCR treatment did not affect plasma glucose concentrations both under fasted condition (Fig. 3a) and during OGTT (Fig. 3e, f) in fructose-fed rats. However, RCR dose-dependently suppressed the increases in basal plasma insulin concentration and the HOMA-IR index (Fig. 3b, c), and had a trend to restore the decreased ratio of glucose to insulin (Fig. 3d).

### Effects on lipid metabolism in rats

**Fig. 4:**
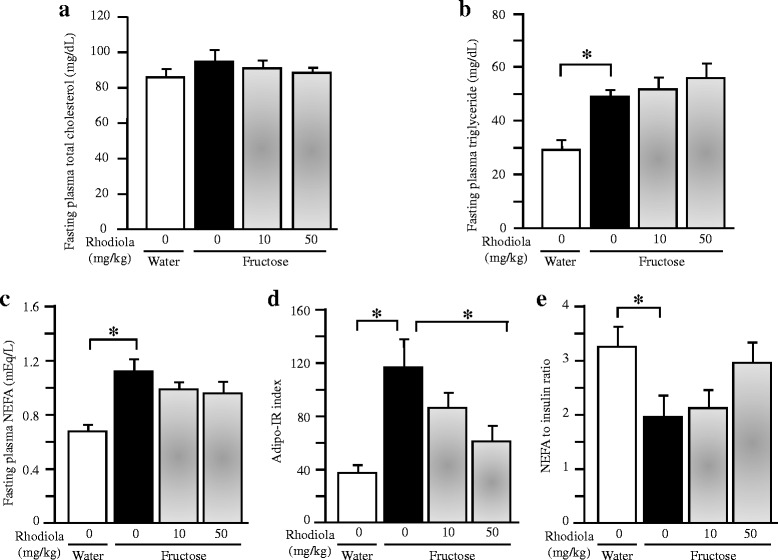
Fasting plasma concentrations of total cholesterol (**a**), triglyceride (**b**) and NEFA (**c**), Adipo-IR index (**d**) and the ratio of NEFA to insulin (**e**) in rats. The fructose controls (*Rhodiola* 0 mg/kg) and fructose *Rhodiola* (50 mg/kg) group had free access to 10 % fructose in their drinking water over 18 weeks. The water controls (*Rhodiola* 0 mg/kg) had free access to a tap water. *Rhodiola* (50 mg/kg) was administered by gavage daily during the last 5 weeks. The water and fructose controls received vehicle (5 % Gum Arabic) alone. Data are means ± SEM (*n* = 6-9 each group). * *P* < 0.05

Fructose feeding did not induce a significant change in plasma total cholesterol concentration (Fig. 4a), but increased plasma triglyceride and NEFA concentrations at the baseline (Fig. 4b, c), and the Adipo-IR index (Fig. 4d). Fructose feeding decreased the ratio of NEFA to insulin (Fig. 4e). RCR treatment minimally affected the fasting plasma concentration of total cholesterol, triglyceride and NEFA (Fig. 4a-c), whereas RCR (50 mg/kg) suppressed the increased Adipo-IR index (Fig. 4d) and had a trend to restore the decreased NEFA to insulin ratio (Fig. 4e).

**Fig. 5:**
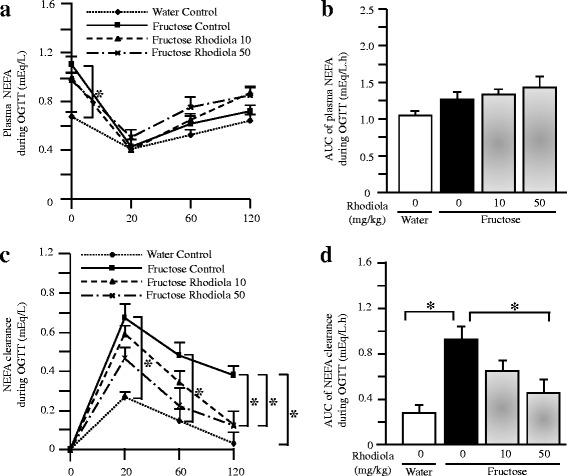
Plasma NEFA concentrations (**a**), the AUC of plasma NEFA concentrations (**b**), plasma NEFA clearance (**c**), and the AUC of plasma NEFA clearance (**d**) during OGTT in rats. The fructose controls (*Rhodiola* 0 mg/kg) and fructose *Rhodiola* (50 mg/kg) group had free access to 10 % fructose in their drinking water over 18 weeks. The water controls (*Rhodiola* 0 mg/kg) had free access to a tap water. *Rhodiola* (50 mg/kg) was administered by gavage daily during the last 5 weeks. The water and fructose controls received vehicle (5 % Gum Arabic) alone. Data are means ± SEM (*n* = 6-9 each group). * *P* < 0.05

Oral glucose administration decreased plasma NEFA concentration in all groups; there was no significant difference in plasma NEFA concentration (Fig. 5a) and the AUC of plasma NEFA concentration (Fig. 5b) during OGTT. However, the clearance of plasma NEFA (Fig. 5c) and the AUC of plasma NEFA clearance (Fig. 5d) after oral glucose administration were increased in fructose controls compared to water controls. RCR treatment did not affect plasma NEFA concentration (Fig. 5a) and the AUC of plasma NEFA concentration (Fig. 5b), but dose-dependently decreased the clearance of plasma NEFA (Fig. 5c) and the AUC of plasma NEFA clearance (Fig. 5d) in fructose-fed rats.

**Fig. 6:**
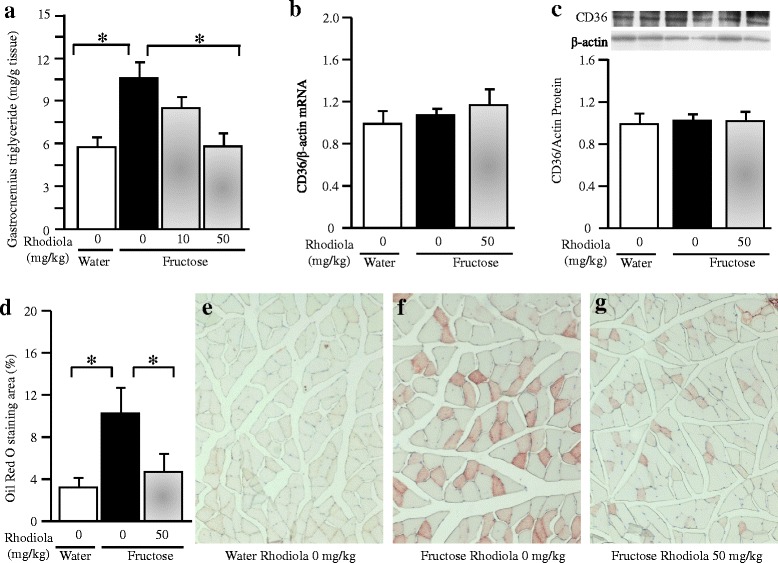
Triglyceride content in the gastrocnemius (**a**), skeletal muscular CD36 mRNA (**b**) and protein (**c**) expression, Oil Red O staining area (**d**-**g**) in rats. The fructose controls (*Rhodiola* 0 mg/kg) and fructose *Rhodiola* (50 mg/kg) group had free access to 10 % fructose in their drinking water over 18 weeks. The water controls (*Rhodiola* 0 mg/kg) had free access to a tap water. *Rhodiola* (50 mg/kg) was administered by gavage daily during the last 5 weeks. The water and fructose controls received vehicle (5 % Gum Arabic) alone. mRNA was determined by Real-time PCR and normalized to β-actin, while protein expression was analyzed by Western blot and normalized to β-actin. Expression in water control was arbitrarily assigned a value of 1. Data are means ± SEM (*n* = 6-9 each group)

Long term fructose consumption also increased triglyceride content (Fig. 6a) and Oil Red O stained area in the gastrocnemius, which was attenuated by RCR treatment (50 mg/kg) (Fig. 6d-g).

### Gene/protein expression profile in rats

As treatment with RCR at 10 mg/kg showed less effect on all parameters observed, gene expression analysis and comparisons were restricted in water control, fructose control and fructose RCR 50 mg/kg groups. By Real-time PCR and Western blot, fructose feeding did not significantly affect total muscular expression of CD36 mRNA and protein; RCR treatment was without effect in fructose-fed rats (Fig. 6b, c).

**Fig. 7:**
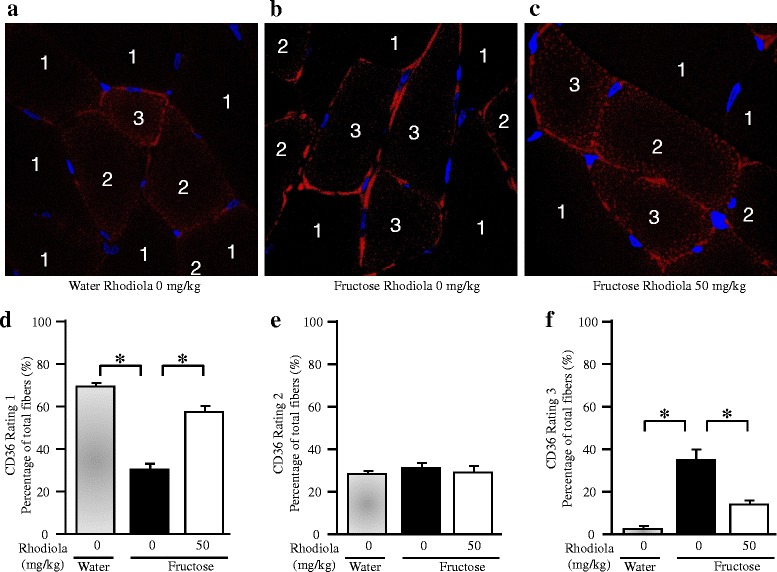
Sarcolemmal CD36 expression in skeletal muscle fibers of rats. The fructose controls (*Rhodiola* 0 mg/kg) and fructose *Rhodiola* (50 mg/kg) group had free access to 10 % fructose in their drinking water over 18 weeks. The water controls (*Rhodiola* 0 mg/kg) had free access to a tap water. *Rhodiola* (50 mg/kg) was administered by gavage daily during the last 5 weeks. The water and fructose controls received vehicle (5 % Gum Arabic) alone. Transverse cryosections from gastrocnemius were fixed and stained with anti-CD36 rabbit polyclonal antibody. Muscle fiber was rated as 1, 2 or 3 based on the intensity of the sarcolemmal CD36 fluorescent signal. ~100 fibers were rated each rat. The percentage of different group fibers given each rating was calculated. Data are means ± SEM (*n* = 6 each group). * *P* < 0.05

Fructose feeding decreased the percentage of fibers expressing sarcolemmal CD36 weakly (rating 1), but increased the percentage of fibers expressing sarcolemmal CD36 abundantly (rating 3), whereas it did not alter the percentage of fibers rated 2 (Fig. 7a-f). RCR treatment completely reversed fructose-induced change in sarcolemmal CD36 expression (Fig. 7a-f).

**Fig. 8:**
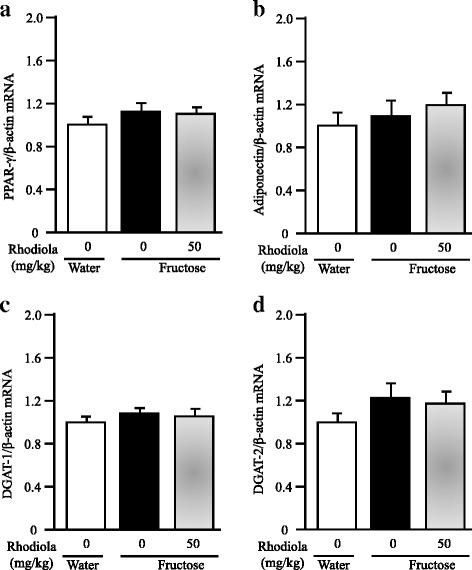
Skeletal muscular mRNA expression of peroxisome proliferator-activated receptor (PPAR)-γ (**a**), adiponectin (**b**), acyl-coenzyme A:diacylglycerol acyltransferase (DGAT)-1 (**c**) and DGAT-2 (**d**) in rats. The fructose controls (*Rhodiola* 0 mg/kg) and fructose *Rhodiola* (50 mg/kg) group had free access to 10 % fructose in their drinking water over 18 weeks. The water controls (*Rhodiola* 0 mg/kg) had free access to a tap water. *Rhodiola* (50 mg/kg) was administered by gavage daily during the last 5 weeks. The water and fructose controls received vehicle (5 % Gum Arabic) alone. mRNA was determined by Real-time PCR and normalized to β-actin. Expression in water control was arbitrarily assigned a value of 1. Data are means ± SEM (*n* = 6-9 each group)

Also in the gastrocnemius, fructose feeding did not alter mRNA expression of peroxisome proliferator-activated receptor (PPAR)-γ (Fig. 8a), adiponectin (Fig. 8b), acyl-coenzyme A:diacylglycerol acyltransferase (DGAT)-1 (Fig. 8c) and DGAT-2 (Fig. 8d). RCR treatment showed minimal effect on expression of these genes (Fig. 8a-d).

## Discussion

The present study clearly demonstrated that treatment with RCR extract suppressed fructose-induced hyperinsulinemia, and increases in the HOMA-IR index and the Adipo-IR index in rats. In addition, the treatment had a trend to restore the ratios of glucose to insulin and NEFA to insulin. These results suggest that RCR extract treatment ameliorates fructose consumption-induced insulin resistance in rats.

Skeletal muscle is regarded as the major site of insulin resistance in obesity and type 2 diabetes [35]. Mounting evidence indicates that insulin resistance is highly associated with excessive intramyocellular triglyceride accumulation [36–38]. High fructose diets are known to cause insulin resistance [39] and triglyceride accumulation [40] in the skeletal muscle of rodents. In healthy subjects with and without a family history of type 2 diabetes, fructose overconsumption also increases lipid accumulation in skeletal muscle [41]. *Rhodiola* species enhance energy levels to increase physical endurance and work productivity [17, 18]. Salidroside, one of the major active components found in RCR, has been demonstrated to stimulate glucose uptake in skeletal muscle cells [42]. These findings suggest that skeletal muscle is one of the major target sites of *Rhodila* species. In the present study, triglyceride accumulation and Oil Red O-stained area in rat skeletal muscle were increased after chronic fructose consumption. Treatment with RCR extract ameliorated the excessive lipid accumulation in fructose-fed rats.

The increased lipid deposition in muscle is secondary to increased fatty acid transport in obese Zucker rats [43], high fat-fed rats [44] and obese, insulin-resistant humans [45]. The increased concentration of blood glucose after oral glucose administration during OGTT strongly stimulates the β-cells to secrete and release insulin, subsequently inhibits adipose lipolysis. Release of fatty acids from adipose tissue under fasted condition is shifted to uptake of fatty acids by key tissues, such as skeletal muscle, after oral glucose administration. Thus, the clearance of plasma fatty acids during OGTT reflects the status of uptake of fatty acids by the key tissues. In the present study, fructose feeding increased clearance of plasma NEFA during OGTT. This acceleration was attenuated after RCR extract treatment. These results suggest that RCR extract treatment may decrease fructose-induced excessive triglyceride accumulation in the skeletal muscle by inhibiting the increased uptake of fatty acids by skeletal muscle.

The fatty acid transport from blood circulation to skeletal muscle involves the translocation of CD36, but not plasma membrane fatty acid–binding protein, from intracellular membrane compartments to the sarcolemma [46]. In muscle from diabetic rodents and humans, more CD36 is recruited to the plasma membrane, leading to persistent enhancement of fatty acid uptake, thereby possibly contributing to the impairment of insulin signaling and glucose utilization [24]. Fructose feeding has been demonstrated to increase CD36 expression in the sarcolemma, but not in whole tissue homogenates from the skeletal muscle, in rats, suggesting a fructose-induced redistribution of this protein associated with fatty acid uptake across the plasma membrane [47]. In the present study, fructose feeding did not alter CD36 expression at the mRNA and protein level in whole skeletal muscle. However, the semi-quantitative assessment by confocal immunofluorescence revealed that sarcolemmal CD36 was overexpressed, while intracellular CD36 distribution in the gastrocnemius was downregulated after fructose feeding. The fructose-induced redistribution of CD36 was restored by RCR extract treatment. Taken together, our findings suggest that RCR extract treatment-elicited insulin-sensitizing action is associated with modulation of CD36 redistribution in the skeletal muscle.

CD36 is a well-known target of PPAR-γ ligands [48]. PPAR-γ agonists stimulate the production of adiponectin, which promotes fatty acid oxidation and insulin sensitivity in muscle and liver [49]. DGATs are the enzymes that catalyze the final step and rate-limiting reaction in triglyceride synthesis. Both DGAT-1 and DGAT-2 in skeletal muscle play a specific role in regulating insulin sensitivity [50]. In the present study, however, fructose feeding did not alter muscular expression of PPAR-γ, adiponectin, DGAT-1 and DGAT-2. RCR extract treatment also showed minimal effect on expression of these genes in fructose-fed rats. Thus, these results do not support an association of modulation of CD36 by RCR extract treatment with muscular PPAR-γ and a link of decreased lipid content to triglyceride synthesis in the skeletal muscle.

Zheng et al. have reported that salidroside ameliorates insulin resistance in db/db mice [22]. The authors also have demonstrated that in vitro salidroside activates the AMP-dependent protein kinase-mediated pathway in the mitochondria of hepatocytes. CD36 and potentially other lipid binding proteins have been demonstrated to function as dynamic regulators of fatty acid transport by relocating from intracellular compartments to the plasma membrane in skeletal muscle in response to pharmacological activation of the AMP-dependent protein kinase by 5-aminoimidazole-4-carboxamide ribonucleoside [51]. This finding suggests a link of CD36 to the AMP-dependent protein kinase. Thus, it needs to further investigate whether modulation of sarcolemmal and intracellular CD36 redistribution in the skeletal muscle by *Rhodiola crenulate* root extract is associated with activation of the muscular AMP-dependent protein kinase-mediated pathway pathway.

Although CD36 is also particularly abundant in adipose tissue, it is still unclear whether CD36 function in adipocytes influences ectopic fat distribution and the pathogenesis of insulin resistance in muscle and liver [25]. In the present study, RCR extract treatment did not affect body weight increase and epididymal fat weight in fructose-fed rats. However, this study still cannot exclude the role of adipose CD36 in RCR extract treatment-elicited amelioration of insulin resistance. On the other hand, we have recently demonstrated that mangiferin, a xanthone glucoside, also mitigates fructose-induced insulin resistance via modulation of CD36 redistribution in the skeletal muscle of Wistar-Kyoto rats [52]. It would be interesting to further investigate whether RCR extract, salidroside, mangiferin and other herb-derived insulin sensitizers share the similar mechanisms to modulate CD36 in ameliorating insulin resistance.

## Conclusions

In conclusion, our present results demonstrate that treatment with RCR extract ameliorates insulin resistance by modulating sarcolemmal and intracellular redistribution in the skeletal muscle of fructose-fed rats. Our findings may provide a better understanding of the traditional use of *Rhodila* species.

## Abbreviations

Adipo-IR, adipose tissue insulin resistance; AUC, the area under the curve; DGAT, acyl-coenzyme A:diacylglycerol acyltransferase; HOMA-IR, the homeostasis model assessment of insulin resistance; NEFA, non-esterified fatty acids; PPAR, peroxisome proliferator-activated receptor; RCR, *Rhodiola crenulata* root.
